# A Unique Case of Midvariant Reverse Takotsubo Cardiomyopathy

**DOI:** 10.1155/2018/2184792

**Published:** 2018-11-21

**Authors:** Carmel Moazez, Vicken Zeitjian, Azar Mehdizadeh

**Affiliations:** Maricopa Integrated Health System, 2601 E Roosevelt St, Phoenix, AZ 85008, USA

## Abstract

Takotsubo cardiomyopathy, also known as stress cardiomyopathy, is known to have 4 variants: apical, midventricular, basal, and focal. Here, we report the 2nd case of reverse midvariant (midventricular) stress cardiomyopathy and the 1st case of reverse midvariant takotsubo cardiomyopathy with apical thrombus.

## 1. Introduction

Stress cardiomyopathy (takotsubo cardiomyopathy), also known as the broken heart syndrome, is a cardiomyopathy which is characterized by transient left ventricular dysfunction in the absence of any coronary artery stenosis. It was first discovered in 1990 by a Japanese cardiologist [1]. Takotsubo is a Japanese word that means a pot with a narrow neck and round bottom that is used to catch octopus [2]. This cardiomyopathy usually occurs in postmenopausal women who undergo severe emotional or physical distress [3].

There are various phenotypes of this syndrome: apical type, basal type (reverse type), midventricular type, and rarely focal type. Here, we report another rare form of stress cardiomyopathy described as reverse midvariant. In this form of stress cardiomyopathy, the mid-left ventricle is hyperdynamic and the apex and the base of the heart are akinetic or hypokinetic. To the best of our knowledge, this is the 2^nd^ case of reverse midvariant stress cardiomyopathy and the 1^st^ case of reverse midvariant cardiomyopathy with apical thrombus reported and described in the literature [4].

## 2. Case Presentation

This is a case of a 59-year-old female with a past medical history significant for chronic obstructive pulmonary disease, prior stress cardiomyopathy with normal coronaries on left heart catheterization and hypertension who presented to the emergency department after being found down by a neighbor. Electrocardiogram (EKG) on admission was significant for sinus tachycardia with a heart rate of 104 beats per minute (bpm) with premature atrial complexes, biatrial enlargement, right axis deviation, and prolonged QT interval. Four minutes later another electrocardiogram was done which showed atrial fibrillation with rapid ventricular rate with a heart rate of 147 bpm and prolonged QT. She was given one dose of IV 5 mg metoprolol and given an amiodarone bolus of 150 mg and started on an amiodarone drip. In light of a CHADSVASC score of 4, she was also started on a heparin drip. Troponin I on admission was found to be 9 ng/ml (normal 0–0.03). She was admitted to the medical intensive care unit.

**Figure 1:**
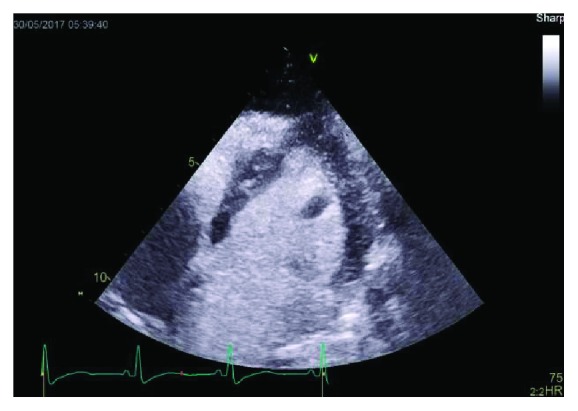
Left ventricular view of the thrombus at the apex of the left ventricle.

**Figure 2:**
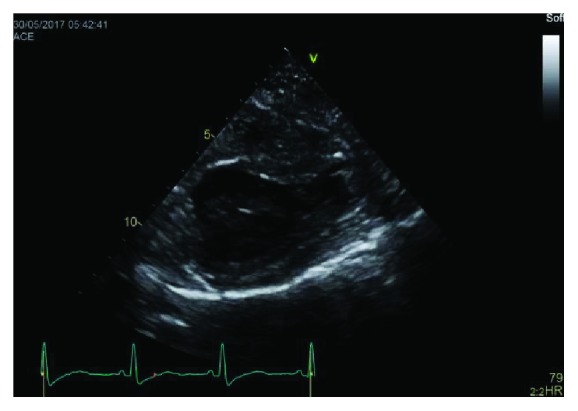
Parasternal short axis showing basal and apical akinesis with preserved contractility of the mid ventricular segments.

**Figure 3 fig3:**
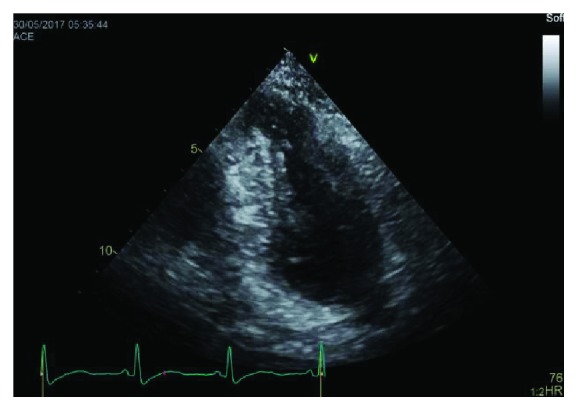
Apical 2 chamber view captured at end systole showing basal and apical akinesis with preserved contractility of the mid ventricular segments.

**Figure 4:**
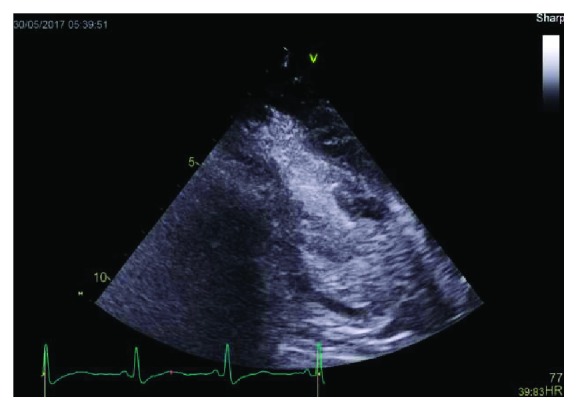
Apical 2 chamber view with agitated saline captured at end systole showing basal and apical ballooning with preserved contractility of the mid ventricular segments.

During this hospitalization her troponin I trended down to 1.5 ng/ml (normal 0–0.03). She had a transthoracic echocardiogram which showed left ventricular ejection fraction 55% (low normal) and a left ventricle apical thrombus measuring 0.95 × 0.7 cm which can be seen in Figure 1. There were also regional wall motion abnormalities which included hypokinetic to akinetic basal myocardial segments, akinetic apex, and hyperdynamic midventricular segments which can be visualized in Figures 2 [Fig fig3]–4. These findings were consistent with reverse midvariant takotsubo cardiomyopathy. She was discharged on aspirin, atorvastatin, metoprolol, and apixaban after 7 days in the hospital. The patient was lost to follow up.

## 3. Discussion

This case describes a rare form of stress cardiomyopathy called reverse midvariant. This phenotype of stress cardiomyopathy can be easily recognized by its unique pattern of hyperdynamic midventricle and akinetic or hypokinetic basal and apical segments. The first reported case of reverse midvariant takotsubo cardiomyopathy was reported in 2016 by Bridgman and Chan. They reported a case of a 79-year-old female who presented with chest pain, elevated troponin I, and a prolonged QT interval with T wave inversion. Echocardiogram was done which showed reverse midvariant takotsubo cardiomyopathy [4]. Our case is unique in that our patient presented with this rare form of stress cardiomyopathy and that her cardiomyopathy was complicated by an apical thrombus.

The pathogenesis of stress cardiomyopathy is unknown. Usually left ventricular dysfunction is transient in stress cardiomyopathy, and many cases have even recovered within a few days [1, 2]. Many experts believe that the catecholamine surge plays a role in wall akinesis. Wittstein et al. showed that patients who presented with takotsubo cardiomyopathy had a two- to threefold increase in catecholamine levels compared with patients who presented with myocardial infarction [5].

Multiple studies have shown the incidence of apical thrombus in takotsubo cardiomyopathy to be between 5–10% [6–8]. Left ventricular thrombi are thought to form during the acute phase of stress cardiomyopathy due to LV akinesis which causes blood stasis and increased catecholamine levels which leads to endothelial injury and hypercoagulable state [6]. Similar to the apical variant of stress cardiomyopathy, this form can also be complicated by apical thrombus as shown in our case. Another possibility is that our patient has been in paroxysmal atrial fibrillation, which manifested this hospitalization and the reason for thrombus formation.

Unfortunately, this patient did not follow up and there were no interim echocardiograms in between acute episodes of stress cardiomyopathy to evaluate left ventricular function. The patient reported compliance with medical therapy with beta blocker and ace inhibitor. The etiology of recurrent and or persistent stress cardiomyopathy in this patient remained unclear. Given recent left heart catheterization and findings of nonobstructed coronaries, the decision was made to not pursue another angiogram.

## 4. Conclusion

We conclude that the reverse midvariant type of stress cardiomyopathy should be added to the existing four forms.

## Supplementary Materials

Supplementary Materials Video 1: apical 2 chamber view with agitated saline showing basal and apical ballooning with preserved contractility of the midventricular segments.
